# Knowledge and Perception on Animal Welfare in Chilean Undergraduate Students with Emphasis on Dairy Cattle

**DOI:** 10.3390/ani11071921

**Published:** 2021-06-28

**Authors:** Einar Vargas-Bello-Pérez, Consuelo Obermöller-Bustamante, Ilona Faber, Tamara Tadich, Paula Toro-Mujica

**Affiliations:** 1 Department of Veterinary and Animal Sciences, University of Copenhagen, Grønnegaårdsvej 3, DK-1870 Frederiksberg C, Denmark; 2 Departamento de Ciencias Animales, Facultad de Agronomía e Ingeniería Forestal, Pontificia Universidad Católica de Chile, Casilla 306, Santiago 6904411, Chile; consuelo.obermoller@outlook.com; 3 Department of Food Science, University of Copenhagen, Rolighedsvej 26, DK-1958 Frederiksberg C, Denmark; ilona.faber@food.ku.dk; 4 Instituto de Ciencia Animal, Facultad de Ciencias Veterinarias, Universidad Austral de Chile, Casilla 567, Valdivia 5090000, Chile; tamara.tadich@uach.cl; 5 Instituto de Ciencias Agroalimentarias, Animales y Ambientales, Universidad de O’Higgins, Ruta 90 km 3, San Fernando 3070000, Chile; paula.toro@uoh.cl

**Keywords:** animal welfare, education, Latin America, future consumers, undergraduate students

## Abstract

**Simple Summary:**

Consumer perceptions on animal welfare have been assessed, providing results that are necessary for the development of policies and legislation regarding animal welfare standards. In Latin America, societal concerns and specifically consumers’ concerns about animal welfare are increasing but so far, the interest of university students on this subject has received little attention. The objectives of this study were to determine differences on knowledge and perception on animal welfare (with emphasis on dairy cattle) from undergraduate students from two universities with different missions and visions and between students from different faculties. Undergraduate students from the two main Chilean universities were surveyed. Overall, this study showed that University only affected the source of animal welfare information used by undergraduate students and some perceptions on dairy cows’ welfare. On the other hand, Faculty did affect most of the variables on awareness, knowledge and perception towards animal welfare. Thus, the knowledge background provided by the faculty of students has a greater influence on their knowledge and perceptions about animal welfare than their university.

**Abstract:**

The objectives of this study were to determine differences in knowledge and perception of animal welfare (with emphasis on dairy cattle) among undergraduate students from two universities with different missions and visions and between students from different faculties. One thousand surveys were obtained from Universidad de Chile (UChile; n = 500) and Pontificia Universidad Católica de Chile (PUC; n = 500) students. The students from both universities were from the following faculties: Agronomy, Architecture, Biology, Economic Sciences, Psychology, Law, Philosophy, Basic Education, Civil Engineering and Medicine. The majority (77%) of students from both universities were aware of animal welfare. Most (56%) students understand animal welfare as the ‘mental and physical state of animals’. Regardless of their faculty, around 97% of the total respondents perceived animal welfare as important for production systems. Regarding specific knowledge about cows’ welfare related to milk management and behavior, students from Economic Sciences, Psychology, Law, Philosophy, Basic Education, and Civil Engineering had less (*p* < 0.001) self-reported knowledge about cows’ basic behavior and specific management practices such as milking and were more negative in their perceptions of dairy production. Overall, results showed that the students’ faculty explained most of the differences among undergraduate students in relation to their perceptions and knowledge about animal welfare. Our data is important, as undergraduate students will make purchasing and power decisions as well as having potential influence on future policies that could modify the animal production industry.

## 1. Introduction

Public interest and concern for animal welfare has increased significantly in recent decades and have led to significant changes such as the development and implementation of the “five freedoms” in developed countries [1,2,3]. Most of the studies carried out point to animal welfare and ethics from a productive perspective in which professionals involved in the field are surveyed, evaluated and interviewed [2,3,4]. 

Consumer perceptions on animal welfare have been assessed, providing results that are necessary for the development of policies and legislation regarding animal welfare standards [5]. In Latin America, societal concerns [6] and specifically consumers’ concerns about animal welfare are increasing [7,8,9] but so far, the interest of university students on this subject has received little attention. Studies focused on undergraduate students are rare and are mostly concentrated in the United States, European Union, Oceania and Africa [3,10,11,12]. This section of society is important since they represent potential future consumers with purchasing and decision-making power, and they may demand animal products that fulfill their quality expectations in aspects such as nutrient quality, but also ethical quality (animal-friendly products). In addition, students could also have a positive influence on policy makers who have the opportunity to shape the animal production industry.

Studies on university students seeking to understand the perception and level of knowledge on animal welfare are limited in Latin America. However, studying this group of the society may deliver different results compared to previously studied groups (consumers and professionals related to animal production). It has been shown that there are significant differences in perception and knowledge about animal welfare between cultures and different countries [13]; therefore, studying differences between students from different educational fields will allow identification of perceptions that may depend on the training and academic field that they have [14]. Sandgren et al. [12] studied undergraduate students and faculty member’s knowledge about animal research, reporting a significant effect of faculty (Biological, Physical and Social Science and Arts and Humanities) on the importance, arguments, and decisions made in relation to animal research.

According to the 2020 QS Word University Rankings [15] provided by Quacquarelli Symonds, the best ranked universities in Chile are Pontificia Universidad Católica de Chile (PUC) and Universidad de Chile (UChile). In terms of numbers of undergraduate students, PUC has around 23,613 [16] whereas UChile has 31,457 [17]. According to the mission and visions, the UChile seeks an integral education that enhances the values of tolerance, diversity and academic excellence [18]. In turn, PUC seeks academic excellence inspired by Catholicism and commitment to the Catholic Church and society [19]. Universities prepare students and their programs to have impacts that later on will be translated into society and culture [20]. Thus, it may be expected that students from these two universities may have different ethical principles. We hypothesized that regarding animal welfare, undergraduate students’ knowledge and perceptions will depend on the type of university as well as the faculty where they are studying. The objectives of this study were firstly, to determine differences on knowledge and perception of animal welfare from undergraduate students from two universities with different missions and visions. Secondly, to determine differences on knowledge and perception of animal welfare from undergraduate students from different faculties. In both objectives, emphasis was made on dairy cattle welfare as we wanted to focus on a specific animal production system and because we have already investigated some of these goals previously in Chilean dairy consumers [8]. Understanding undergraduate student’s knowledge and perception on animal welfare is important, as they will have purchase and power decision and a potential influence on future policies that could modify the animal production industry.

## 2. Materials and Methods

This study was approved by the Scientific Ethical Committee on Social Sciences, Arts and Humanities of the Pontificia Universidad Católica de Chile (ID 170302001).

### 2.1. Data Collection and Participants

One thousand surveys were obtained between both universities: 500 students from UChile and 500 from PUC. The following faculties were included: Agronomy, Architecture, Biology, Economic Sciences, Psychology, Law, Philosophy, Basic Education, Civil Engineering, and Medicine, since these faculties are present in both universities. Fifty questionnaires were collected from each faculty. Similar sample sizes were targeted to make comparisons between faculties and between universities.

In order to apply face-to-face surveys seven pollsters were trained on the specific terminologies and contents about animal welfare and dairy cow’s welfare. Pollsters were undergraduate students that had gone through lectures on basic concepts about animal welfare. Pollsters were trained to obtain clear answers from the students or in case of clarifications. After training, between March and May 2016, pollsters performed surveys from Monday to Friday between 12:00 and 15:00 h (around lunchtime), and randomly selected students that were outside classrooms. Pollsters only surveyed students that voluntarily wanted to participate in the survey. All of them provided verbal consent in order to participate. The response rate per day was 5 to 10 answered surveys. Students were not previously recruited and no personal information such as name and student ID were obtained to ensure anonymity, and thus, no signed consent was requested. All respondents were 18 years of age or older. Survey responses were recorded in SurveyMonkey. 

### 2.2. Questionnaire Development

The questionnaire had 29 closed-ended questions (six were dichotomous) and was divided into the following sections: demographic characteristics, general knowledge on animal welfare, specific questions about dairy cow’s welfare, and perception on animal welfare and different commodities. Questions related to dairy science were made as this is the main research line in our group and we have previously assessed this topic within consumers [8]. The full questionnaire can be found as Supplementary Material. Before using the final questionnaire, a pilot survey was performed among 30 individuals to evaluate the questions on clarity, accuracy of response options, usage of scientific terminology and the overall flow of the survey. The questions used for this study were inspired or adapted from similar surveys [3,7,12,13,21].

### 2.3. Statistical Analysis

To calculate total scores of overall knowledge of animal welfare, variables about animal welfare knowledge from section two of the questionnaire (except question 8, 9, 10, 11, 12, 17 and 21) were coded into 1 for “yes” or “true” answers and 0 for “no” or “I don’t know” answers. Those scores were used in the following answers: awareness of five freedoms (Yes = 1), awareness of the Law on protection of animals in Chile (Yes = 1), animals have emotions (Yes = 1), animals have personality types (Yes = 1), anesthetics are used in castration (Yes = 1), calf separated from mother after birth (True = 1) and dehorning in bovine dairies (Is done for ease of handling = 1). Scores for overall knowledge of animal welfare were computed by the sum of the latter mentioned variables. The overall scores generated a maximum score ranging from 0 to 7. Regarding self-reported knowledge, the response categories ‘Low knowledge’ and ‘No knowledge’ were combined into the category ‘Low/No ’ due to few responses. 

Descriptive statistics are shown as frequencies and percentages for categorical data and as mean and standard deviation (SD) for total scores of knowledge of animal welfare. Two-way ANOVA was performed to find possible differences in total knowledge scores regarding animal welfare between students from different faculties and PUC and UChile. An ordinal logistic regression analysis on the association between self-reported knowledge and total scores of knowledge was performed with adjustment for age, gender, grade, faculty and university. Chi-square tests for independence and Fisher’s exact tests were used to identify possible differences among students from different faculties, universities, age groups, gender and grade in knowledge of animal welfare (e.g., scores of overall knowledge, levels of self-reported knowledge) and perceptions towards animal welfare (e.g., quality of life in animal production systems, highest level of animal welfare under certain conditions). Post hoc tests with Bonferroni corrections for multiple comparisons was conducted on significant values to discover specific differences across groups of faculties, age, gender and grade. *p*-values of <0.05 were considered statistically significant. All statistical analyses were performed in RStudio version 1.2.5042 [22]. 

## 3. Results

### 3.1. Respondents Characteristics

A total of 1000 students from PUC and UChile completed the survey. Table 1 shows the respondents’ characteristics from both universities. The PUC sample included slightly more male respondents, whereas slightly more women were included in the UChile sample (<0.001). An equal distribution of students from different faculties from both universities was obtained. Most respondents were aged between 18 and 23 years.

### 3.2. Knowledge about Animal Welfare

Table 2 shows awareness and knowledge of animal welfare among students from PUC and UChile. Most students from PUC (75.8%) and UChile (77.2%) were aware of animal welfare. Knowledge scores (scale 0 to 7) did not differ significantly (*p* = 0.420) between the two universities and was on average 3.7 for PUC students and 3.8 for UChile students. About half of the PUC (49.4%) and UChile (52.8%) students indicated high self-reported knowledge of animal welfare but this was not significant (*p* = 0.400). Significant differences (*p* = 0.012) were found in the source of information used to gain knowledge of animal welfare between students from PUC and UChile; however, overall, most PUC (31.8%) and UChile (35.0%) students use social media channels. 

More PUC students (14.2%) gain knowledge via educational institutions than UChile students (9.6%) while more UChile students (18%) than PUC students (12.6%) gain knowledge via family and friends (*p* = 0.012). Most PUC (56.2%) and UChile students (54.8%) stated animal welfare as ‘mental and physical state of animals’ but this was not significant (*p* = 0.758). Only a minority of students across different faculties (4.1%) indicated that animal welfare involves ‘avoiding the use of animals to meet the needs and requirements of humans’. Soil was noted as preferable over concrete for dairy cows to walk on indicated by the majority of PUC (83.2%) and UChile (86.0%) students (*p* = 0.076). A high interest in animal welfare courses was shown by students from PUC (84.2%) and UChile (85.2%) but this was not significant (*p* = 0.725). 

Table 3 shows awareness and knowledge of animal welfare across different faculties. Although awareness of animal welfare was high overall among PUC and UChile students, significant differences were found across the faculties (*p* < 0.001). Significantly, more Administration and Commerce students (92%) stated awareness of animal welfare compared to Art and Architecture (71%), Health (70%), Humanity (69%) and Technology (67%) students. Total score of knowledge about animal welfare was on average 4.5 out of 7 and significantly higher among Agriculture students (4.51 out of 7) compared to other faculties (except Exact and Natural Sciences) (*p* < 0.001). Conversely, only 36% of Agriculture students and 29% of Exact and Natural science students indicated a high self-reported knowledge of animal welfare. However, significantly more Law (64%), Education (64%) and Art and Architecture (62%) students than Agriculture and Exact and Natural science students stated their knowledge regarding animal welfare to be high (*p* < 0.001). Significantly more Education (44%) and Technology (44%) students compared to Exact and Natural science students (19%) use social media apps to gain knowledge of animal welfare, whilst significantly more Agriculture (43%) and Exact and Natural science (36%) students compared to the other faculties indicated to gain knowledge of animal welfare via educational institutions (*p* < 0.001).

Among the Social Science students, 76% stated animal welfare as the physical and mental health of animals, while Art and Architecture students (46%), Humanity students (37%) and Administration and Commerce students (38%) indicated this (*p* < 0.001). Most students from Humanity (59%), and Administration and Commerce (60%) indicated ‘appropriate policies and legislation’ as related to animal welfare. Furthermore, nearly all students stated that soil is most comfortable than concrete for dairy cows to walk on. While slightly fewer Administration and commerce students (78%) stated this (*p* = 0.003), the post hoc test did not show significant differences across the faculties. Although 84.7% of the students across different faculties are interested in following an animal welfare course, this was significantly lower among Art and Architecture students (55%) compared to the other faculties (except Agriculture) (*p* < 0.001). 

Additional ordinal logistic regression analysis on the association between self-reported knowledge and total scores of perceived knowledge did not show a significant association (OR = 1.10, 95% CI −0.01–0.19, *p*-value = 0.09) (Table S1).

### 3.3. Perception of Animal Welfare

Table 4 presents the perceptions toward animal welfare among PUC and UChile students. Nearly all students from PUC (97.2%) and UChile (97.6%) perceived animal welfare as important for productive systems (*p* = 0.843). Regarding quality of life (QoL) in different production systems, significantly (*p* = 0.005) more UChile students (24.8%) than PUC students (17.4%) perceived the production system of cow’s milk as poor. However, 25.4% of the UChile students and 25.6% of the PUC students perceived the system of cow’s milk production as good (*p* = 1.000). Only a minority of the students from PUC (4.6%) and UChile (2.8%) perceived the salmon production system as good (*p* = 0.180), and 24.6% of the PUC students and 26.2% of the UChile students perceived this system as poor (*p* = 0.611).

Most surveyed students from PUC (45.2%) and UChile (49.2%) perceived the ‘rearing’ process as the most important operation in animal production, whereas ‘transportation’ and ‘slaughter’ was stated by only 3.4% and 7.6% of the PUC respectively and 2.2% and 6.2% of the UChile students respectively (*p* = 0.438). More than half of the students from PUC (56.2%) and UChile (52.4%) perceived food products that consider animal welfare during production as tastier (*p* = 0.354). In terms of nutritional quality, 60.6% of the PUC students and 59.6% of the UChile students indicated ‘higher nutritional quality’ when food products consider animal welfare (*p* = 0.916). 

Table 5 presents the perceptions toward animal welfare across different faculties. No significant differences (*p* = 0.158) were found in perceived importance of animal welfare for productive systems across the faculties, as nearly all students stated this as important. Differences were found in perception toward QoL in different production systems across the faculties. More (*p* < 0.001) Agriculture (38%) and Social Science (36%) students compared to Exact and Natural Science students (15%) perceived QoL in cow’s milk production as good. At the same time, many Exact and natural science students (35%) perceived this as poor. 

The QoL in beef production was perceived as good by significantly more Art and architecture (36%) and Agriculture (40%) students compared to Health students (10%), while this was indicated as poor by 38% of the Humanity students. Significantly more Law students (44%) compared to Exact and Natural Science (15%), Humanities (16%), Technology (20%) and Health (15%) students indicated QoL in salmon (*Salmo salar*) production as poor (*p* < 0.001). Only a minority of the Agriculture (8%) students stated QoL in pig (*Sus scrofa*) production as good, while this was stated by significantly more students from the other faculties (except Social Science and Education students, *p* < 0.001). QoL in laying hens’ (*Gallus gallus*) production was reported as good by significantly more Education (37%), Administration and Commerce (30%) and Health (23%) students compared to Agriculture students (6%) (*p* < 0.001), while many Agriculture students (39%) indicated it as poor.

Significantly more Education (59%) students compared to Agriculture students (32%) perceived ‘rearing’ as an important operation in animal production (*p* < 0.001). The proportion of students perceiving ‘food’ as an important operation was significantly higher in the faculty of Law (32%) and Agriculture (31%) compared to Social Science (5%). Only a minority of the students across different faculties perceived transportation and slaughter to be an important operation. Significantly more Social Science students (84%) and Administration and Commerce students (75%) compared to other faculties (except Art and Architecture (62%) and Law (55%)) perceived food products that consider animal welfare during production as tastier, this was lowest among Humanity (29%) students (*p* < 0.001). In terms of nutrition quality, significantly more Art and Architecture (81%) and Social science (90%) students compared to other faculties (except Technology (66%)) stated that food products that consider animal welfare have ‘higher nutritional quality’ (*p* < 0.001).

Table 6 shows the perceptions towards animal welfare in dairy cows of students from PUC and UChile. Significant differences between universities were only found in the alternatives associated with the dairy milking process. Most students stated that higher levels of animal welfare could be achieved when dairy cows are in a grazing production or when a mixture of in-door and grazing production is present. Only a minority of students stated that in-door production gives the highest level of animal welfare. Maternal behavior was perceived as most important behavior by dairy cows among most surveyed students. Furthermore, significantly more PUC (57.2%) students than UChile students (48.6%) stated that milking a dairy cow causes the cow stress (*p* = 0.015).

Table 7 shows results of students’ perceptions toward animal welfare in dairy cows across different faculties. Significantly more students from faculty of Art and Architecture (62%) compared to Health students (26%) considered ‘mixture of in-door and grazing’ to give the highest level of animal welfare in dairy (*p* < 0.001). Only a minority of the students considered in-door production or that ‘animals must be free’ to give the highest level of animal welfare. Furthermore, significantly, more Health (57%) students compared to Art and Architecture (33%), Law (33%) and Social Science (29%) students considered grazing production to give the highest level of animal welfare. Significantly fewer students from the faculty of Art and Architecture (39%) compared to Exact and Natural Sciences (75%), Humanities (72%), Technology (78%) and Education (78%) perceived maternal behavior as the most important behavior for dairy cows (*p* < 0.001). Significantly, fewer Agriculture students (38%) compared to Social Science (65%) and Health (64%) students stated that milking a dairy cow causes the cow stress (*p* < 0.001). 

In terms of perceptions toward animal welfare in general and in dairy cows across different sociodemographic factors, most perceptions did not differ significantly across age groups, gender and grade (Supplementary Materials, Tables S2–S7). Nevertheless, the proportion of students aged 24–26 (36.9%) stating QoL in beef production as good was significantly higher compared to students aged 18–20 (19.5%) and 21–23 (20.4%) (*p* < 0.001) (Table S2). Furthermore, significantly fewer students aged 18–20 (11.9%) compared to students aged 21–23 (21.6%) and >26 (28.2%) considered grooming as the most important behavior performed by dairy cows, while significantly more students aged 18–20 (69.6%) considered maternal behavior as important (*p* < 0.001). The proportion of students aged 21–23 (13.0%) stating that when milking, a dairy cow has pleasure was significantly lower compared to the other three age groups (*p* < 0.001) (Table S3). Significantly more female respondents (26.3%) compared to male respondents (18.5%) considered QoL in beef production as good (*p* = 0.004). The proportion of male respondents (25.5%) considering QoL in laying hens production as good was significantly larger compared to the proportion of female respondents (17.1%) stating this (*p* = 0.001). Furthermore, significantly more male respondents (63.9%) compared to female respondents (56.2%) stated that food products made while taking into account animal welfare have higher nutritional quality (*p* = 0.003) (Table S4). Significantly more male respondents (66.8%) compared to female respondents (53.6%) stated maternal behavior as the most important behavior among dairy cows (*p* < 0.001) (Table S5). A significantly larger proportion of Sixth grade students (37.8%) compared to First (17.1%), Second (20.6%) and Fourth (19.5%) grade students considered QoL in beef production as good ((*p* = 0.004), while significantly more First grade students (26.5%) considered it as poor (*p* = 0.025). QoL in pig production was perceived as good by significantly fewer Sixth grade students (8.9%) compared to First (31.8%), Second (37.3%) and Fourth (29.3%) grade students (*p* < 0.001) (Table S6). A larger proportion of Sixth grade students (63.3%) compared to First (33.5%), Second (44.5%), Third (40.6%) and Fifth (42.0%) grade students considered the highest level of animal welfare in a mixture of in-door and grazing (*p* = 0.002) (Table S7). 

## 4. Discussion

Perceptions and attitudes of different stakeholders towards animal welfare have been widely assessed, with most studies focusing on consumers [7,8,9], animal sciences [13], veterinary science [3,21] and agricultural sciences students [23,24] and farmers [14,25], with only one study, focusing specifically on the use of animals in research, including students from different fields of knowledge [12,26]. In the present study, a novel perspective is provided by trying to understand if the university (mission and vision) could influence students’ perceptions on this matter, as well as their faculty.

When searching for differences between universities, a significant association (*p*= 0.012) was found between university of origin and the source of information used by students to acquire information relative to animal welfare. Students from PUC preferred social media apps, followed by websites, educational institutions and then family and friends, while students from UChile had the same first two preferences, but more students chose family and friends as a source of animal welfare information, and educational institutions were the least chosen source. According to CADEM [27] after interviewing over 1700 participants in Chile, most people check their social media apps constantly during the day (at least once per hour); being WhatsApp, Facebook, Instagram, YouTube and Twitter the most used apps. Thus, it is not surprising that social media apps are used as the source of information; young adults tend to engage more in sharing and discussing animal welfare issues [28] in platforms such as Twitter, Instagram and Facebook. 

On the other hand, non-profit animal welfare organizations work with small budgets, making social media attractive as a means of sharing their contents [29]. This network of messages referring to an identical subject has been described as “hashtag activism” [30]. Many hashtags are associated to animal welfare issues and are used to raise awareness and promote discussion. For example, Wonneberger et al. [31] studied the dynamics in Twitter of animal welfare hashtags related to relevant cases such as the over-fed chicken and the kilo-stunner, both issues generated discussion by different stakeholders. Having social media apps as primary source of information could also be related to the high percentage of self-reported awareness about animal welfare found (overall 76.5%). It has been reported that people having a primary source of information that is used by animal protection groups to highlight animal welfare issues had significantly higher concern and awareness of animal welfare [32]. However, information from sources such as these may be biased against the production industry. It is also important mentioning that this information has the potential to be biased as in any other extremist platform.

The self-reported knowledge on animal welfare did not differ between universities, with most students reporting a high level of knowledge, and 18.1% reporting a low level of knowledge or no interest in the subject. Animal welfare is a societal concern; with increasing interest in the subject during the last decades [33], thus it was expected to have low percentages of participants not showing interest. Nevertheless, a significant difference was found between faculties of origin where a lower percentage of students from Exact and Natural Science and Agricultural Science reported high levels of self-reported knowledge, although they obtained the highest mean scores of knowledge. Contrary to our results, studies comparing self-reported knowledge with actual knowledge report overconfidence of undergraduates [34]. Thus, more information on how confidence is enhanced as students develop in their careers would be needed, since confidence is a significant part of learning with confident students learning better [35], this considering that students from Agricultural education areas report educational institutions as their main source of information about animal welfare.

In terms of perception of welfare according to productive system, a significant difference was only observed between universities for the quality of life of dairy cows, with a higher percentage of undergraduates from UChile considering it poor. No differences between the two universities were found for the most important operations involved in these production systems, organoleptic and nutritional quality animal-friendly products. Interestingly, significant differences did appear when comparing the same variables across faculties, indicating that it is not particularly the University vision that influences attitudes and perceptions of undergraduate students on animal welfare. However, this study did not record personal information such as background and interests of participants, which may also be affecting results. The role of individual background in perceptions of animal welfare deserves further attention.

Significant differences between faculties were found, with lower levels of awareness about animal welfare in students from Technology and Humanities, and the highest level of awareness was reported for students from Administration and Commerce, which was even higher than for those from Agricultural Sciences. This was unexpected, since in agricultural science students their perceptions could be influenced by their knowledge about production systems and animals, but at the same time the increasing number of labelling systems, willingness to pay more for, and added value given to animal friendly products in Chile [7] could be a new area of interest for those undergoing Administration and Commerce studies. A positive finding is that, except for Humanities and Administration and Commerce, students of all other faculties understood animal welfare as the adequate mental and physical state of an animal, allowing the expression of their natural behaviors.

In this study, students from faculties such as Agriculture and Health, where biological sciences are taught, had positive perceptions towards dairy, beef and pig production systems. This finding agrees in part with Balschweid [23] who evaluated perceptions of high school students focused on agricultural science on animal production and reported that more than 80% of the surveyed students agreed that raising animals for food is a noble profession or activity. Regarding salmon production, faculties related to social sciences were more negative and perceived poor animal welfare in that production system. Here, it is important to note that in Chile, salmon production is one of the fastest-growing industries that has developed with very limited regulations, and that has resulted in significant effects on the ecosystem and local communities [36]. Therefore, it may be expected to observe negative expectations from those surveyed students where social events are important in their education. 

In general, results between faculties about animal welfare agrees with Gaworski and Turbakiewicz [26]. The authors studied the perception of undergraduate and graduate students from different faculties (Humanities, Medical, Economic, Art and Life Sciences studies) on animal welfare. They found that most students, regardless of their faculty, perceived animal welfare as a pivotal factor to keep high production standards and comfort in livestock animals.

In the specific case of dairy cows, in this study, students from PUC and UChile expressed that, higher levels of animal welfare could be achieved when dairy cows are in a grazing production system or when there is a mixture of in-door and grazing. This is somehow explained by the fact that in Chile, due to its geographical conditions, most dairy farms are based on grazing systems [7] where animals are out-doors and Chilean society normally associates grazing with milk production [8]. 

With regard to specific knowledge on cow’s welfare related to milk management and behaviour, it is clear that students that are closer to social sciences, technology, administration and commerce have less knowledge on basic cows’ behavior and specific managements such as milking and are more negative in their perceptions towards dairy production. Signal et al. [37] reported that attitudes to animals and empathy relate to school-based humane education programs in a surveyed Australian community. They also reported a link between human-directed empathy and attitudes to animals and the category or type of animal being considered affects the strength of that link.

In relation to the strengths and limitations of the present study it has to be considered that a face-to-face survey was applied to 1000 students; half from Universidad de Chile (UChile) and the other half from Pontificia Universidad Católica de Chile (PUC), within these universities, 50 students per faculty were surveyed. 

Face-to-face surveys provide better data quality than web-based surveys, although they are more time consuming and expensive to apply [38]. One of the disadvantages of face-to-face surveys is social desirability, which tends to be higher for this method, but in the present study, students were recruited and trained to apply the survey. Having an interviewer of the same age and a university student could encourage the development of trust, reducing social desirability [39]. A second bias could be associated to participants balanced distribution according to gender and year of study; although it was expected to have a greater response rate from women. It has been reported that women tend to engage more with surveys and other activities involving exchange of information [21,40]. This gender balance could be the result of sampling bias, since the magnitude of observed differences between demographic groups is less pronounced in face-to-face surveys compared to online ones [41]. Finally, it also needs to be considered that response rate was not registered. Internationally there has been a reduction in response rates to face-to-face surveys, being refusal to respond the main reason [42]. In the present study the data collection effort was to obtain 100 surveys per career, this was reached by obtaining between 5–10 surveys daily, but the total number of contacts was not registered. Underreporting of refusals is one of the main problems when trying to understand nonresponse to face-to-face surveys [43] and something that needs to be improved in future studies. 

To our knowledge, this is the first study determining knowledge, perception and opinions from undergraduate students from the best-ranked universities of Chile. Our study provides an initial “picture” from this subject and allowed to visualize how further research efforts could establish more insights with a more specific framework and perhaps focusing on specific groups of undergraduate students. 

## 5. Conclusions

This study showed that universities (with different missions and visions) only affected the source of animal welfare information used by undergraduate students and some perceptions on dairy cows’ welfare. On the other hand, faculty did affect most of the variables studied. Thus, faculties have a greater influence on their knowledge and perceptions about animal welfare than their university. Understanding undergraduate student’s knowledge and perception on animal welfare is essential, as they will have purchase and power decision as well as potential impacts on future policies that could modify the animal production industry.

## Figures and Tables

**Table 1:** Characteristics of respondents studying Pontificia Universidad Católica de Chile (PUC) and Universidad de Chile (UChile).

	Participants, No. (%)			
	Total (n = 1000)	PUC (n = 500)	UChile (n = 500)	*p*-Value
*Age*				0.475 ^1^
18–20 years	428 (42.8)	212 (42.4)	216 (43.2)	
21–23 years	422 (42.2)	220 (44.0)	202 (40.4)	
24–26 years	111 (11.1)	52 (10.4)	59 (11.8)	
More than 26 years	39 (3.9)	16 (3.2)	23 (4.6)	
*Gender*				<0.001 ^1^
Male	509 (50.9)	282 (56.4)	227 (45.4)	
Female	491 (49.1)	218 (43.6)	273 (54.6)	
*Faculty*				1.000 ^1^
Agricultural	100 (10.0)	50 (10.0)	50 (10.0)	
Art and architecture	100 (10.0)	50 (10.0)	50 (10.0)	
Exact and natural sciences	100 (10.0)	50 (10.0)	50 (10.0)	
Social Sciences	100 (10.0)	50 (10.0)	50 (10.0)	
Law	100 (10.0)	50 (10.0)	50 (10.0)	
Humanities	100 (10.0)	50 (10.0)	50 (10.0)	
Education	100 (10.0)	50 (10.0)	50 (10.0)	
Technology	100 (10.0)	50 (10.0)	50 (10.0)	
Health	100 (10.0)	50 (10.0)	50 (10.0)	
Administration and commerce	100 (10.0)	50 (10.0)	50 (10.0)	
*Year of study*				0.4055 ^1^
First	170 (17.0)	84 (16.8)	86 (17.2)	
Second	209 (20.9)	102 (20.4)	107 (21.4)	
Third	207 (20.7)	99 (19.8)	108 (21.6)	
Fourth	174 (17.4)	87 (17.4)	87 (17.4)	
Fifth	150 (15.0)	87 (17.4)	63 (12.6)	
Sixth	90 (9.0)	41 (8.2)	49 (9.8)	

^1^ Chi-square test for independence.

**Table 2:** Awareness and knowledge of animal welfare of students from Pontificia Universidad Católica de Chile (PUC) and Universidad de Chile (UChile).

	Total (n = 1000)	PUC (n = 500)	UChile (n = 500)	*p*-Value
Awareness of animal welfare, n (%)	765 (76.5)	379 (75.8)	386 (77.2)	0.655 ^1^
Total scores of knowledge (0–7), mean (SD) ^4^	4.50 (1.37)	3.7 (1.35)	3.8 (1.15)	0.420 ^2^
*Self-reported level of knowledge*				0.400 ^3^
High, n (%)	511 (51.1)	247 (49.4)	264 (52.8)	
Medium, n (%)	307 (30.7)	153 (30.6)	154 (30.8)	
Low/No, n (%) ^5^	178 (17.8)	97 (19.4)	81 (16.2)	
Not interested, n (%)	4 (0.4)	3 (0.6)	1 (0.2)	
*Source of information*				0.012 ^1^
Educational institutions, n (%)	119 (11.9)	71 (14.2)	48 (9.6)	
Social media apps, n (%)	334 (33.4)	159 (31.8)	175 (35.0)	
Friends and family, n (%)	153 (15.3)	63 (12.6)	90 (18.0)	
Internet (e.g., websites), n (%)	208 (20.8)	100 (20.0)	108 (21.6)	
Mass media (e.g., radio, television, cinema), n (%)	65 (6.5)	36 (7.2)	29 (5.8)	
None, I’ve never heard about animal welfare, n (%)	121 (12.1)	71 (14.2)	50 (10.0)	
*Understanding of animal welfare*				0.758 ^1^
Mental and physical state, n (%)	555 (55.5)	281 (56.2)	274 (54.8)	
Appropriate policies and legislation, n (%)	404 (40.4)	197 (39.4)	207 (41.4)	
Avoid use of animals, n (%)	41 (4.1)	22 (4.4)	19 (3.8)	
*Dairy cows feel more comfortable when walking on:*				0.076 ^1^
Smooth cement, n (%)	25 (2.5)	18 (3.6)	7 (1.4)	
Soil, n (%)	846 (84.6)	416 (83.2)	430 (86.0)	
I don’t know, n (%)	129 (12.9)	66 (13.2)	63 (12.6)	
Interested in animal welfare course, n (%)	847 (84.7)	421 (84.2)	426 (85.2)	0.725 ^1^

^1^ Chi-square test for independence. ^2^ One-way ANOVA. ^3^ Fisher’s exact test. ^4^ Total scores of knowledge are derived from the sum of response of the following variables: awareness of five freedoms (Yes = 1), awareness of Law on protection of animals in Chile (Yes = 1), animals have emotions (Yes = 1), animals have personality types (Yes = 1), anaesthetics is used in castration (Yes = 1), calf separated from mother after birth (Yes = 1), and dehorning is done with ease of handling (Yes = 1). ^5^ ‘Low/no’ is a combined category consisting of responses to ‘Low knowledge’ and ‘No knowledge’.

**Table 3:** Awareness and knowledge of animal welfare of Chilean university students from different faculties.

	Agricultura (n = 100)	Art and Architecture (n = 100)	Exact and Natural Sciences (n = 100)	Social Sciences (n = 100)	Law (n = 100)	Humanities (n = 100)	Education (n = 100)	Technology (n = 100)	Health (n = 100)	Administration and Commerce (n = 100)	*p*-Value
Awareness of animal welfare, n (%)	84 (84.0) ^a,b^	71 (71.0) ^b^	80 (80.0) ^a,b^	79 (79.0) ^a,b^	77 (77.0) ^a,b^	69 (69.0) ^b^	76 (76.0) ^a,b^	67 (67.0) ^b^	70 (70.0) ^b^	92 (92.0) ^a^	<0.001 ^1^
Total scores of knowledge (0–7), mean (SD) ^4^	4.51 (1.37) ^a^	3.73 (1.28) ^b,c^	4.04 (1.18) ^a,b^	3.64 (1.05) ^b,c^	3.41 (1.24) ^c^	3.45 (1.38) ^c^	3.64 (1.00) ^b,c^	3.62 (1.10) ^b,c^	3.63 (1.33) ^b,c^	3.67 (1.22) ^b,c^	<0.001 ^2^
*Self-reported level of knowledge*											<0.001 ^3^
High, n (%)	36 (36.0) ^a,b^	62 (62.0) ^c^	29 (29.0) ^b^	57 (57.0) ^a,c^	64 (64.0) ^c^	49 (49.0) ^a,b,c^	64 (64.0) ^c^	51 (51.0) ^a,b,c^	53 (53.0) ^a,c^	46 (46.0) ^a,b,c^	
Medium, n (%)	46 (46.0) ^a,b^	36 (36.0) ^a,b,c^	52 (52.0) ^b^	22 (22.0) ^c,d^	12 (12.0) ^d^	29 (29.0) ^a,c,d^	27 (27.0) ^a,c,d^	34 (34.0) ^a,b,c^	26 (26.0) ^a,c,d^	23 (23.0) ^c,d^	
Low/No, n (%) ^5^	18 (18.0) ^a,b^	2 (2.0) ^c^	19 (19.0) ^a,b^	21 (21.0) ^a,b^	23 (23.0) ^a,b^	21 (21.0) ^a,b^	9 (9.0) ^b,c^	13 (13.0) ^a,b,c^	21 (21.0) ^a,b^	31 (31.0) ^a^	
Not interested, n (%)	0 (0) ^a^	0 (0) ^a^	0 (0) ^a^	0 (0) ^a^	1 (1) ^a^	1 (1) ^a^	0 (0) ^a^	2 (2) ^a^	0 (0) ^a^	0 (0) ^a^	
*Source of information*											<0.001 ^1^
Educational institutions, n (%)	43 (43.0) ^a^	6 (6.0) ^b^	36 (36.0) ^a^	3 (3.0) ^b^	5 (5.0) ^b^	10 (10.0) ^b^	3 (3.0) ^b^	3 (3.0) ^b^	8 (8.0) ^b^	2 (2.0) ^b^	
Social media channels, n (%)	26 (26.0) ^a,b^	35 (35.0) ^a,b^	19 (19.0) ^b^	24 (24.0) ^a,b^	38 (38.0) ^a,b^	31 (31.0) ^a,b^	44 (44.0) ^a^	44 (44.0) ^a^	38 (38.0) ^a,b^	35 (35.0) ^a,b^	
Friends and family, n (%)	17 (17.0) ^a^	12 (12.0) ^a,b^	14 (14.0) ^a,b^	27 (27.0) ^b^	16 (16.0) ^a,b^	7 (7.0) ^a^	15 (15.0) ^a,b^	17 (17.0) ^a,b^	19 (19.0) ^a,b^	9 (9.0) ^a^	
Internet (e.g., websites), n (%)	10 (10.0) ^a^	42 (42.0) ^b^	19 (19.0) ^a,c^	21 (21.0) ^a,b,c^	21 (21.0) ^a,b,c^	33 (33.0) ^b,c^	18 (18.0) ^a,c^	19 (19.0) ^a,c^	11 (11.0) ^a^	14 (14.0) ^a,c^	
Mass media (e.g., radio, television, cinema), n (%)	3 (3.0) ^a^	4 (4.0) ^a^	4 (4.0) ^a^	5 (5.0) ^a^	11 (11.0) ^a^	3 (3.0) ^a^	13 (13.0) ^a^	7 (7.0) ^a^	6 (6.0) ^a^	9 (9.0) ^a^	
None, I’ve never heard about animal welfare, n (%)	1 (1.0) ^a^	1 (1.0) ^a^	8 (8.0) ^a,b^	20 (20.0) ^b,c^	9 (9.0) ^a,b^	16 (16.0) ^b,c^	7 (7.0) ^a,b^	10 (10.0) ^a,b^	18 (18.0) ^b,c^	31 (31.0) ^c^	
*Understanding of animal welfare*											<0.001 ^3^
Mental and physical state, n (%)	62 (62.0) ^a,b,c^	46 (46.0) ^c,d,e^	60 (60.0) ^a,b,c,d,e^	76 (76.0) ^b^	52 (52.0) ^a,c,d,e^	37 (37.0) ^e^	56 (56.0) ^a,b,c,d,e^	61 (61.0) ^a,b,c,d^	67 (67.0) ^a,b,c^	38 (38.0) ^d,e^	
Appropriate policies and legislation, n (%)	34 (34.0) ^a,b,c,d,e^	43 (43.0) ^d,e,f,g,h^	40 (40.0) ^c,e,f,g,h^	17 (17.0) ^b^	47 (47.0) ^a,c,d,e,f,g,h^	59 (59.0) ^g,h^	36 (36.0) ^a,b,c,d,e,h^	37 (37.0) ^a,b,c,d,e,f,g,h^	31 (31.0) ^a,b,c,d,e^	60 (60.0) ^f,g^	
Avoid use of animals, n (%)	4 (4.0) ^a,b^	11 (11.0) ^b^	0 (0.0) ^a^	7 (7.0) ^a,b^	1 (1.0)	4 (4.0) ^a,b^	8 (8.0) ^a,b^	2 (2.0) ^a,b^	2 (2.0) ^a,b^	2 (2.0) ^a,b^	
*Dairy cows feel more comfortable when walking on:*											0.003 ^3^
Smooth cement, n (%)	6 (6.0) ^a^	6 (6.0) ^a^	3 (3.0) ^a^	1 (1.0) ^a^	3 (3.0) ^a^	0 (0.0) ^a^	1 (1.0) ^a^	3 (3.0) ^a^	0 (0.0) ^a^	2 (2.0) ^a^	
Soil, n (%)	81 (81.0) ^a^	87 (87.0) ^a^	83 (83.0) ^a^	89 (89.0) ^a^	84 (84.0) ^a^	82 (82.0) ^a^	92 (92.0) ^a^	90 (90.0) ^a^	80 (80.0) ^a^	78 (78.0) ^a^	
I don’t know, n (%)	13 (13.0) ^a^	7 (7.0) ^a^	14 (14.0) ^a^	10 (10.0) ^a^	13 (13.0) ^a^	18 (18.0) ^a^	7 (7.0) ^a^	7 (7.0) ^a^	20 (20.0) ^a^	20 (20.0) ^a^	
Interested in animal welfare course, n (%)	73.0 (73.0) ^a,b^	55 (55.0) ^b^	94 (94.0) ^c^	92 (92.0) ^c^	93 (93.0) ^c^	91 (91.0) ^c^	86 (86.0) ^a,c^	93 (93.0) ^c^	88 (88.0) ^a,c^	82 (82.0) ^a,c^	<0.001 ^1^

^1^ Chi-square test for independence and posthoc test with Bonferroni corrections for multiple comparisons. Different superscript letters indicate a significant difference. ^2^ One-way ANOVA with Bonferroni corrections for multiple comparisons. Different superscript letters indicate a significant difference. ^3^ Fisher’s exact test and posthoc test with Bonferroni corrections for multiple comparisons. Different superscript letters indicate a significant difference. ^4^ Total scores of knowledge are derived from the sum of response of the following variables: awareness of five freedoms (Yes = 1), awareness of Law on protection of animals in Chile (Yes = 1), animals have emotions (Yes = 1), animals have personality types (Yes = 1), anaesthetics is used in castration (Yes = 1), calf separated from mother after birth (Yes = 1), and dehorning is done with ease of handling (Yes = 1). ^5^ ‘Low/no’ is a combined category consisting of responses to ‘Low knowledge’ and ‘No knowledge’.

**Table 4:** Perceptions toward animal welfare of student from Pontificia Universidad Católica de Chile (PUC) and Universidad de Chile (UChile).

	Total (n = 1000)	PUC (n = 500)	UChile (n = 500)	*p*-Value
Importance of animal welfare in productive system, n (%)	974 (97.4)	486 (97.2)	488 (97.6)	0.843 ^1^
*Quality of life in production of:*				
Cow’s milk				
Good, n (%)	255 (25.5)	128 (25.6)	127 (25.4)	1.000 ^1^
Poor, n (%)	211 (21.1)	87 (17.4)	124 (24.8)	0.005 ^1^
Beef				
Good, n (%)	223 (22.3)	108 (21.6)	115 (23.0)	0.649 ^1^
Poor, n (%)	206 (20.6)	110 (22.0)	96 (19.2)	0.309 ^1^
Salmon				
Good, n (%)	37 (3.7)	23 (4.6)	14 (2.8)	0.180 ^1^
Poor, n (%)	254 (25.4)	123 (24.6)	131 (26.2)	0.611 ^1^
Pig				
Good, n (%)	271 (27.1)	133 (26.6)	138 (27.6)	0.776 ^1^
Poor, n (%)	89 (8.9)	53 (10.6)	36 (7.2)	0.076 ^1^
Laying hens				
Good, n (%)	214 (21.4)	108 (21.6)	106 (21.2)	0.939 ^1^
Poor, n (%)	240 (24.0)	127 (25.4)	113 (22.6)	0.336 ^1^
*Most important operations in animal production*				0.438 ^1^
Food, n (%)	206 (20.6)	100 (20.0)	106 (21.2)	
Transportation, n (%)	28 (2.8)	17 (3.4)	11 (2.2)	
Slaughter, n (%)	69 (6.9)	38 (7.6)	31 (6.2)	
Rearing, n (%)	472 (47.2)	226 (45.2)	246 (49.2)	
Accommodation, n (%)	225 (22.5)	119 (23.8)	106 (21.2)	
*Food products made while taking into account animal welfare compared to products that do not take this into account*				0.354 ^1^
Better flavour, n (%)	543 (54.3)	281 (56.2)	262 (52.4)	
Same flavour, n (%)	186 (18.6)	85 (17.0)	101 (20.2)	
I don’t know, n (%)	271 (27.1)	134 (26.8)	137 (27.4)	
*Food products made while taking into account animal welfare compared to products that do not take this into account*				0.916 ^1^
Higher nutritional quality, n (%)	601 (60.1)	303 (60.6)	298 (59.6)	
Same nutritional quality, n (%)	187 (18.7)	91 (18.2)	96 (19.2)	
I don’t know, n (%)	212 (21.2)	106 (21.2)	106 (21.2)	

^1^ Chi-square test for independence.

**Table 5:** Perceptions toward animal welfare of Chilean university students from different faculties.

	Agricultural n = 100)	Art and Architecture (n = 100)	Exact and Natural Sciences (n = 100)	Social Sciences (n = 100)	Law (n = 100)	Humanities (n = 100)	Education (n = 100)	Technology (n = 100)	Health (n = 100)	Administration and Commerce (n = 100)	*p*-Value
Importance of animal welfare in productive system, n (%)	99 (99.0)	99 (99.0)	99 (99.0)	97 (97.0)	99 (99.0)	97 (97.0)	97 (97.0)	98 (98.0)	92 (92.0)	97 (97.0)	0.158 ^1^
*Quality of life in production of:*											
Cow’s milk											
Good, n (%)	38 (38.0) ^a^	17 (17.0) ^b,c^	15 (15.0) ^c^	36 (36.0) ^a,b^	22 (22.0) ^a,b,c^	28 (28.0) ^a,b,c^	21 (21.0) ^a,b,c^	21 (21.0) ^a,b,c^	32 (32.0) ^a,b,c^	25 (25.0) ^a,b,c^	<0.001 ^2^
Poor, n (%)	10 (10.0) ^a^	19 (19.0) ^a,b,c^	35 (35.0) ^c^	29 (29.0) ^b,c^	19 (19.0) ^a,b,c^	17 (17.0) ^a,b,c^	13 (13.0) ^a,b^	26 (26.0) ^a,b,c^	25 (25.0) ^a,b,c^	18 (18.0) ^a,b,c^	<0.001 ^2^
Beef											
Good, n (%)	40 (40.0) ^a^	36 (36.0) ^a,b^	22 (22.0) ^a,b,c,d^	27 (27.0) ^a,b,d^	21 (21.0) ^a,b,c,d^	9 (9.0) ^c^	19 (19.0) ^a,b,c,d^	21 (21.0) ^a,b,c,d^	10 (10.0) ^c,d^	18 (18.0) ^b,c,d^	<0.001 ^2^
Poor, n (%)	16 (16.0) ^a^	11 (11.0) ^a^	12 (12.0) ^a^	13 (13.0) ^a^	16 (16.0) ^a^	38 (38.0) ^b^	28 (28.0) ^a,b^	20 (20.0) ^a,b^	25 (25.0) ^a,b^	27 (27.0) ^a,b^	<0.001 ^2^
Salmon											
Good, n (%)	8 (8.0)	2 (2.0)	5 (5.0)	2 (2.0)	2 (2.0)	6 (6.0)	2 (2.0)	4 (4.0)	5 (5.0)	1 (1.0)	0.200 ^1^
Poor, n (%)	23 (23.0) ^a,b,c^	23 (23.0) ^a,b,c^	15 (15.0) ^c^	34 (34.0) ^a,b,c^	44 (44.0) ^b^	16 (16.0) ^a,c^	31 (31.0) ^a,b,c^	20 (20.0) ^a,c^	15 (15.0) ^a,c^	33 (33.0) ^a,b,c^	<0.001 ^2^
Pig											
Good, n (%)	8 (8.0) ^a^	33 (33.0) ^b^	39 (39.0) ^b^	13 (13.0) ^a,c^	33 (33.0) ^b^	35 (35.0) ^b^	21 (21.0) ^a,b,c^	33 (33.0) ^b^	30 (30.0) ^b,c^	26 (26.0) ^b,c^	<0.001 ^2^
Poor, n (%)	12 (12.0) ^a,b^	20 (20.0) ^b^	11 (11.0) ^a,b^	13 (13.0) ^a,b^	4 (4.0) ^a^	5 (5.0) ^a,b^	4 (4.0) ^a^	5 (5.0) ^a,b^	6 (6.0) ^a,b^	9 (9.0) ^a,b^	<0.001 ^2^
Laying hens											
Good, n (%)	6 (6.0) ^a^	12 (12.0) ^a,b^	19 (19.0) ^a,b,c^	22 (22.0) ^a,b,c^	22 (22.0) ^a,b,c^	22 (22.0) ^a,b,c^	37 (37.0) ^c^	21 (21.0) ^a,b,c^	23 (23.0) ^b,c^	30 (30.0) ^b,c^	<0.001 ^2^
Poor, n (%)	39 (39.0) ^a^	27 (27.0) ^a,b^	27 (27.0) ^a,b^	11 (11.0) ^b^	17 (17.0) ^b^	24 (24.0) ^a,b^	24 (24.0) ^a,b^	29 (29.0) ^a,b^	29 (29.0) ^a,b^	13 (13.0) ^b^	<0.001 ^2^
*Most important operations in animal production*											<0.001 ^1^
Food, n (%)	31 (31.0) ^a^	21 (21.0) ^a,b^	19 (19.0) ^a,b,c^	5 (5.0) ^c^	32 (32.0) ^a^	22 (22.0) ^a,b^	19 (19.0) ^a,b,c^	24 (24.0) ^a,b^	22 (22.0) ^a,b^	11 (11.0) ^b,c^	
Transportation, n (%)	16 (16.0) ^a^	3 (3.0) ^a,b^	0 (0.0) ^b^	2 (2.0) ^b^	0 (0.0) ^b^	0 (0.0) ^b^	2 (2.0) ^b^	0 (0.0) ^b^	0 (0.0) ^b^	5 (5.0) ^a,b^	
Slaughter, n (%)	6 (6.0) ^a^	6 (6.0) ^a^	10 (10.0) ^a^	4 (4.0) ^a^	10 (10.0) ^a^	4 (4.0) ^a^	8 (8.0) ^a^	9 (9.0) ^a^	10 (10.0) ^a^	2 (2.0) ^a^	
Rearing, n (%)	32 (32.0) ^a^	37 (37.0) ^a,b^	51 (51.0) ^a,b^	47 (47.0) ^a,b^	45 (45.0) ^a,b^	53 (53.0) ^a,b^	59 (59.0) ^b^	51 (51.0) ^a,b^	54 (54.0) ^a,b^	43 (43.0) ^a,b^	
Accommodation, n (%)	15 (15.0) ^a,b^	33 (33.0) ^b,c,d,e^	20 (20.0) ^a,b,e^	42 (42.0) ^d^	13 (13.0) ^a^	21 (21.0) ^a,b,c,d,e^	12 (12.0) ^a^	16 (16.0) ^a,b^	14 (14.0) ^a,b^	39 (39.0) ^c,d,e^	
*Food products made while taking into account animal welfare compared to products that do not take this into account, have:*											<0.001 ^2^
Better flavor, n (%)	52 (52.0) ^a,b^	62 (62.0) ^b,c^	47 (47.0) ^a,b,d^	84 (84.0) ^e^	55 (55.0) ^a,b,c^	29 (29.0) ^d^	52 (52.0) ^a,b^	51 (51.0) ^a,b,d^	36 (36.0) ^a,d^	75 (75.0) ^c,e^	
Same flavour, n (%)	32 (32.0) ^a^	21 (21.0) ^a,b^	25 (25.0) ^a,b^	9 (9.0) ^b^	16 (16.0) ^a,b^	9 (9.0) ^b^	24 (24.0) ^a,b^	21 (21.0) ^a,b^	21 (21.0) ^a,b^	8 (8.0) ^b^	
I don’t know, n (%)	16 (16.0) ^a,b,c^	17 (17.0) ^a,b,c^	28 (28.0) ^c,d^	7 (7.0) ^b^	29 (29.0) ^a,c,d^	62 (62.0) ^e^	24 (24.0) ^a,c,d^	28 (28.0) ^a,c,d^	43 (43.0) ^d,e^	17 (17.0) ^a,b,c^	
*Food products made while taking into account animal welfare compared to products that do not take this into account, have:*											<0.001 ^2^
Higher nutritional quality, n (%)	54 (54.0) ^a^	81 (81.0) ^b,c^	55 (55.0) ^a^	90 (90.0) ^c^	50 (50.0) ^a^	46 (46.0) ^a^	56 (56.0) ^a^	66 (66.0) ^a,b^	49 (49.0) ^a^	54 (54.0) ^a^	
Same nutritional quality, n (%)	32 (32.0) ^a^	13 (13.0) ^a,b,c,d,e,f^	16 (16.0) ^a,b,c,d,e,f^	7 (7.0) ^e,f^	24 (24.0) ^a,d^	7 (7.0) ^c,f^	27 (27.0) ^a,b,d^	10 (10.0) ^b,c,d,e,f^	20 (20.0) ^a,b,c,d,e,f^	31 (31.0) ^a^	
I don’t know, n (%)	14 (14.0) ^a,b,c,d,e^	6 (6.0) ^d,e^	29 (29.0) ^c,f^	3 (3.0) ^b,e^	26 (26.0) ^a,c,f^	47 (57.0) ^f^	17 (17.0) ^a,c,d^	24 (24.0) ^a,c^	31 (31.0) ^a,c,f^	15 (15.0) ^a,b,c,d,e^	

^1^ Fisher’s exact test and posthoc test with Bonferroni corrections for multiple comparisons. Different superscript letters indicate a significant difference. ^2^ Chi-square test for independence and posthoc test with Bonferroni corrections for multiple comparisons. Different superscript letters indicate a significant difference.

**Table 6:** Chilean university students’ perceptions toward dairy cows’ welfare.

	Total (n = 1000)	PUC (n = 500)	UChile (n = 500)	*p*-Value
*Highest level of animal welfare for dairy cows in:*				0.163 ^1^
In-door production, n (%)	18 (1.8)	10 (2.0)	8 (1.6)	
Grazing production, n (%)	421 (42.1)	193 (38.6)	22.8 (45.6)	
Mixture of in-door and grazing, n (%)	438 (43.8)	233 (46.6)	205 (41.0)	
None, animals must be free, n (%)	123 (12.3)	64 (12.8)	59 (11.8)	
*Most important behavior performed by dairy cows:*				0.211 ^1^
Grooming, n (%)	176 (17.6)	98 (19.6)	78 (15.6)	
Maternal behavior, n (%)	603 (60.3)	287 (57.4)	316 (63.2)	
Reproduction, n (%)	95 (9.5)	52 (10.4)	43 (8.6)	
Game behavior, n (%)	126 (12.6)	63 (12.6)	63 (12.6)	
*When milking, a dairy cow:*				0.015 ^1^
Gets stressed, n (%)	529 (52.9)	286 (57.2)	243 (48.6)	
Is suffering, n (%)	102 (10.2)	42 (8.4)	60 (12.0)	
Is in pain, n (%)	176 (17.6)	75 (15.0)	101 (20.2)	
Has pleasure, n (%)	193 (19.3)	97 (19.4)	96 (19.2)	

Pontificia Universidad Católica de Chile (PUC) and Universidad de Chile (UChile). ^1^ Chi-square test for independence.

**Table 7:** Chilean university students’ perceptions toward dairy cows’ welfare.

	Agricultural (n = 100)	Art and Architecture (n = 100)	Exact and Natural Sciences (n = 100)	Social Sciences (n = 100)	Law (n = 100)	Humanities (n = 100)	Education (n = 100)	Technology (n = 100)	Health (n = 100)	Administration and Commerce (n = 100)	*p*-Value
*Highest level of animal welfare for dairy cows in:*											<0.001 ^1^
In-door production, n (%)	1 (1.0) ^a^	3 (3.0) ^a^	0 (0.0) ^a^	3 (3.0) ^a^	6 (6.0) ^a^	1 (1.0) ^a^	2 (2.0) ^a^	1 (1.0) ^a^	1 (1.0) ^a^	0 (0.0) ^a^	
Grazing production, n (%)	47 (47.0)	33 (33.0) ^a^	52 (52.0)	29 (29.0) ^a^	33 (33.0) ^a^	34 (34.0)	35 (35.0)	52 (52.0)	57 (57.0) ^b^	49 (49.0)	
Mixture of in-door and grazing, n (%)	52 (52.0) ^a,b^	62 (62.0) ^b^	38 (38.0) ^a,c^	50 (50.0) ^a,b^	47 (47.0) ^a,b,c^	38 (38.0) ^a,c^	43 (43.0) ^a,b,c^	36 (36.0) ^a,c^	26 (26.0) ^c^	46 (46.0) ^a,b,c^	
None, animals must be free, n (%)	0 (0.0) ^a^	2 (2.0) ^a,b^	10 (10.0) ^a,b,c,d^	18 (18.0) ^c,d^	14 (14.0) ^b,c,d^	27 (27.0) ^d^	20 (20.0) ^c,d^	11 (11.0) ^b,c,d^	16 (16.0) ^c,d^	5 (5.0) ^a,b,c^	
*Most important behavior performed by dairy cows:*											<0.001 ^2^
Grooming, n (%)	28 (28.0) ^a,b^	30 (30.0) ^b^	6 (6.0) ^c^	13 (13.0) ^a,b,c^	26 (26.0) ^a,b^	19 (19.0) ^a,b,c^	18 (18.0) ^a,b,c^	7 (7.0) ^c^	10 (10.0) ^a,c^	19 (19.0) ^a,b,c^	
Maternal behavior, n (%)	42 (42.0) ^a,b^	39 (39.0) ^b^	75 (75.0) ^c,d^	42 (42.0) ^a,b^	60 (60.0) ^a,b,c,d^	72 (72.0) ^c,d^	63 (63.0) ^a,c,d^	78 (78.0) ^d^	78 (78.0) ^d^	54 (54.0) ^a,b,c^	
Reproduction, n (%)	21 (21.0) ^a,b^	24 (24.0) ^b^	5 (5.0) ^c^	10 (10.0) ^a,b,c^	5 (5.0) ^c^	1 (1.0) ^c^	9 (9.0) ^a,b,c^	7 (7.0) ^a,c^	8 (8.0) ^a,b,c^	5 (5.0) ^c^	
Game behavior, n (%)	9 (9.0) ^a,b^	7 (7.0) ^a,b^	14 (14.0) ^a,b^	35 (35.0) ^c^	9 (9.0) ^a,b^	8 (8.0) ^a,b^	10 (10.0) ^a,b^	8 (8.0) ^a,b^	4 (4.0) ^b^	22 (22.0) ^a,c^	
*When milking, a dairy cow:*											<0.001 ^2^
Gets stressed, n (%)	38 (38.0) ^a^	59 (59.0) ^a,b,c^	55 (55.0) ^a,b,c^	65 (65.0) ^c^	48 (48.0) ^a,b,c^	52 (52.0) ^a,b,c^	58 (58.0) ^a,b,c^	49 (49.0) ^a,b,c^	64 (64.0) ^b,c^	41 (41.0) ^a,b^	
Is suffering, n (%)	16 (16.0) ^a^	12 (12.0) ^a^	14 (14.0) ^a^	8 (8.0) ^a^	6 (6.0) ^a^	10 (10.0) ^a^	9 (9.0) ^a^	13 (13.0) ^a^	5 (5.0) ^a^	9 (9.0) ^a^	
Is in pain, n (%)	20 (20.0) ^a^	14 (14.0) ^a^	15 (15.0) ^a^	21 (21.0) ^a^	14 (14.0) ^a^	19 (19.0) ^a^	20 (20.0) ^a^	24 (24.0) ^a^	14 (14.0) ^a^	15 (15.0) ^a^	
Has pleasure, n (%)	26 (26.0) ^a,b^	15 (15.0) ^b,c^	16 (16.0) ^a,b,c^	6 (6.0) ^c^	32 (32.0) ^a,b^	19 (19.0) ^a,b,c^	13 (13.0) ^b,c^	14 (14.0) ^b,c^	17 (17.0) ^a,b,c^	35 (35.0) ^a^	

^1^ Fisher’s exact test and posthoc test with Bonferroni corrections for multiple comparisons. Different superscript letters indicate a significant difference; ^2^ Chi-square test for independence and posthoc test with Bonferroni corrections for multiple comparisons. Different superscript letters indicate a significant difference.

## Data Availability

Data is available online at http://doi.org/10.5281/zenodo.4930884 (accessed on 24 April 2021).

## Supplementary Materials

The following are available online at https://www.mdpi.com/article/10.3390/ani11071921/s1, Table S1: Association between self-reported knowledge and total knoweldge scores of animal welfare of students from Pontificia Universidad Católica de Chile (PUC) and Universidad de Chile (UChile). Table S2: Chilean university students’ perceptions toward animal welfare stratified by age groups. Table S3: Chilean university students’ perceptions toward dairy cows welfare stratified by age groups. Table S4: Chilean university students’ perceptions toward animal welfare stratified by gender. Table S5: Chilean university students’ perceptions toward dairy cows welfare stratified by gender. Table S6: Chilean university students’ perceptions toward animal welfare stratified by grade. Table S7: Chilean university students’ perceptions toward dairy cows welfare stratified by grade. Full survey.
