# Supplementary material for: Knowledge and Perception on Animal Welfare in Chilean Undergraduate Students with Emphasis on Dairy Cattle
Source: Animals (Basel). 2021 Jun 28;11(7):1921. doi: 10.3390/ani11071921 (PMC8300360; doi:10.3390/ani11071921)
Supplement: Supplementary file 1 [file animals-11-01921-s001.zip › animals-1219543-supplementary.pdf]

Table S1. Association between self-reported knowledge and total knoweldge scores of animal welfare of students from Pontificia Universidad Católica de Chile (PUC) and Universidad de Chile (UChile)

Independent Variables/Covariates	Total (n = 996)[1]		
		95% CI	
	OR[2]	Lower	Upper
Total knowledge score (0-7)	1.10	-0.01	0.19
Age[3]			
21 – 23	0.73	-0.70	0.05
24 – 26	1.15	-0.42	0.69
27 and older	0.98	-0.80	0.74
Male	0.95	-0.23	0.14
Grade[4]			
Second	0.67	-0.81	0.00
Third	0.72	-0.09	0.76
Fourth	0.84	-0.35	0.70
Fifth	0.71	-0.90	0.22
Sixth	1.24	-0.43	0.88
University of Chile	0.84	-0.42	0.07
Faculty[5]			
Art and architecture	1.10	-0.29	0.49
Exact and natural sciences	1.49	0.00	0.80
Social Sciences	1.53 *	0.04	0.81
Law	1.20	-0.22	0.57
Humanities	0.64 *	-0.85	-0.05
Education	1.40	-0.05	0.73
Technology	0.62 *	-0.87	-0.10
Health	1.66 *	0.10	0.91
Administration and commerce	1.45	-0.04	0.78

OR = odds ratios, CI = confidence interval, * $p < 0.05$, ** < 0.01, *** $p < 0.001$

[1] Excludes participants who responded with ´not interested´ to the question regarding self-reported knowledge of animal welfare

[2] Ordinal logistic regression

[3] Reference group is age group ´18-20´

[4] Reference group is first grade

[5] Reference group is faculty of Agriculture

Table S2. Chilean university students´ perceptions toward animal welfare stratified by age groups.

	**18-20** (n = 428)	**21-23** (n = 422)	**24-26** (n = 111)	**> 26** (n = 39)	P-value
Importance of animal welfare in productive system, n (%)	417 (97.4)	410 (97.2)	109 (98.2)	38 (97.4)	0.971[1]
Quality of life in production of:					
Cow´s milk					
Good, n (%)	99 (23.1)	115 (27.3)	28 (25.2)	13 (33.3)	0.360[2]
Poor, n (%)	100 (23.4)	89 (21.1)	19 (17.1)	3 (7.7)	0.362[1]
Beef					
Good, n (%)	84 (19.6)[a]	86 (20.4)[a]	41 (36.9)[b]	12 (30.8)[a,b]	<0.001[1]
Poor, n (%)	95 (22.2)	86 (20.4)	19 (17.1)	6 (15.4)	0.364[2]
Salmon					
Good, n (%)	16 (3.7)	13 (3.1)	6 (5.4)	2 (5.1)	0.537[1]
Poor, n (%)	114 (26.6)	105 (24.9)	23 (20.7)	12 (30.8)	0.363[2]
Pig					
Good, n (%)	132 (30.8) [a]	115 (27.3) [a,b]	19 (17.1) [b]	5 (12.8) [a,b]	0.004[1]
Poor, n (%)	29 (6.8)	43 (10.2)	13 (11.7)	4 (10.3)	0.373[1]
Laying hens					
Good, n (%)	97 (22.7)	93 (22.0)	17 (15.3)	7 (17.9)	0.355[2]
Poor, n (%)	90 (21.0)	99 (23.5)	37 (33.3)	14 (35.9)	0.358[2]
Most important operations in animal production					0.025[1]
Food, n (%)	74 (17.3) [a]	91 (21.6) [a]	30 (27.0) [a]	11 (28.2) [a]	
Transportation, n (%)	13 (3.0) [a]	8 (1.9) [a]	5 (4.5) [a]	2 (5.1) [a]	
Slaughter, n (%)	29 (6.8) [a]	33 (7.8) [a]	4 (3.6) [a]	3 (7.7) [a]	
Rearing, n (%)	195 (45.6) [a]	213 (50.5) [a]	47 (42.3) [a]	17 (43.6) [a]	
Accommodation, n (%)	117 (27.3) [a]	77 (18.2) [b]	25 (22.5) [a,b]	6 (15.4) [a,b]	
Food products made while taking into account animal welfare compared to products that do not take this into account					0.022[2]
Better flavour, n (%)	247 (57.7) [a]	222 (52.6) [a]	57 (51.4) [a]	17 (43.6) [a]	
Same flavour, n (%)	69 (16.1) [a]	77 (18.2) [a]	25 (22.5) [a,b]	15 (38.5) [b]	
I don´t know, n (%)	112 (26.2) [a]	123 (29.1) [a]	29 (26.1) [a]	7 (17.9) [a]	
Food products made while taking into account animal welfare compared to products that do not take this into account					0.026[2]
Higher nutritional quality, n (%)	244 (57.0) [a]	269 (63.7) [a]	67 (60.4) [a]	21 (53.8)[a]	
Same nutritional quality, n (%)	94 (22.0) [a]	58 (13.7)[b]	23 (20.7)[a,b]	12 (30.8) [a]	
I don´t know, n (%)	90 (21.0) [a]	95 (22.5) [a]	21 (18.9) [a]	6 (15.4) [a]	

[1] Fisher´s exact test and posthoc test with Bonferroni corrections for multiple comparisons.
Different superscript letters indicate a significant difference.
[2] Chi-square test for independence and posthoc test with Bonferroni corrections for multiple comparisons.
Different superscript letters indicate a significant difference.

Table S3. Chilean university students´ perceptions toward dairy cows welfare stratified by age groups.

	**18-20** (n = 428)	**21-23** (n = 422)	**24-26** (n = 111)	**> 26** (n = 39)	P-value
Highest level of animal welfare for dairy cows in:					0.212[1]
In-door production, n (%)	8 (1.9)	7 (1.7)	2 (1.8)	1 (2.6)	
Grazing production, n (%)	187 (43.7)	180 (42.7)	42 (37.8)	12 (30.8)	
Mixture of in-door and grazing, n (%)	174 (40.7)	182 (43.1)	58 (52.3)	24 (61.5)	
None, animals must be free, n (%)	59 (13.8)	53 (12.6)	9 (8.1)	2 (5.1)	
Most important behavior performed by dairy cows:					<0.001[1]
Grooming, n (%)	51 (11.9) [a]	91 (21.6) [b]	23 (20.7) [a,b]	11 (28.2) [b]	
Maternal behavior, n (%)	298 (69.6) [a]	223 (52.8) [b]	57 (51.4) [b]	25 (64.1) [a,b]	
Reproduction, n (%)	34 (7.9) [a]	48 (11.4) [a]	13 (11.7) [a]	0 (0.0) [a]	
Game behavior, n (%)	45 (10.5) [a]	60 (14.2) [a]	18 (16.2) [a]	3 (7.7) [a]	
When milking, a dairy cow:					<0.001[1]
Gets stressed, n (%)	207 (48.4) [a]	237 (56.2) [a]	63 (56.8) [a]	22 (56.4) [a]	
Is suffering, n (%)	49 (11.4) [a]	49 (11.6) [a]	3 (2.7) [b]	1 (2.6) [a,b]	
Is in pain, n (%)	75 (17.5) [a]	81 (19.2) [a]	18 (16.2) [a]	2 (5.1) [a]	
Has pleasure, n (%)	97 (22.7) [a]	55 (13.0) [b]	27 (24.3) [a]	14 (35.9) [a]	

[1] Fisher´s exact test and posthoc test with Bonferroni corrections for multiple comparisons. Different superscript letters indicate a significant difference.

Table S4. Chilean university students´ perceptions toward animal welfare stratified by gender.

	**Female** (n = 491)	**Male** (n = 509)	P-value
Importance of animal welfare in productive system, n (%)	473 (96.3)	501 (98.4)	0.037[1]
Quality of life in production of:			
Cow´s milk			
Good, n (%)	118 (24.0)	137 (26.9)	0.331[1]
Poor, n (%)	105 (21.4)	106 (20.8)	0.871[1]
Beef			
Good, n (%)	129 (26.3)	94 (18.5)	0.004[1]
Poor, n (%)	88 (17.9)	118 (23.2)	0.044[1]
Salmon			
Good, n (%)	20 (4.1)	17 (3.3)	0.607[1]
Poor, n (%)	115 (23.4)	139 (27.3)	0.159[1]
Pig			
Good, n (%)	140 (28.5)	131 (25.7)	0.354[1]
Poor, n (%)	56 (11.4)	33 (6.5)	0.005[1]
Laying hens			
Good, n (%)2	84 (17.1)	130 (25.5)	0.001[1]
Poor, n (%)	127 (25.9)	113 (22.2)	0.194[1]
Most important operations in animal production			0.083[1]
Food, n (%)	108 (22.0)	98 (19.3)	
Transportation, n (%)	13 (2.9)	15 (2.9)	
Slaughter, n (%)	41 (5.5)	28 (5.5)	
Rearing, n (%)	234 (46.8)	238 (46.8)	
Accommodation, n (%)	95 (25.5)	130 (25.5)	
Food products made while taking into account animal welfare compared to products that do not take this into account			0.082[1]
Better flavour, n (%)	250 (50.9)	293 (57.6)	
Same flavour, n (%)	102 (20.8)	84 (16.5)	
I don´t know, n (%)	139 (28.3)	132 (25.9)	
Food products made while taking into account animal welfare compared to products that do not take this into account			0.003[1]
Higher nutritional quality, n (%)	276 (56.2)	325 (63.9)	
Same nutritional quality, n (%)	89 (18.1)	98 (19.3)	
I don´t know, n (%)	126 (25.7)	86 (16.9)	

[1] Chi-square test for independence

Table S5. Chilean university students´ perceptions toward dairy cows welfare stratified by gender.

	**Female** (n = 491)	**Male** (n = 509)	P-value
Highest level of animal welfare for dairy cows in:			<0.001[1]
In-door production, n (%)	11 (2.2)	7 (1.4)	
Grazing production, n (%)	212 (43.2)	209 (41.1)	
Mixture of in-door and grazing, n (%)	230 (46.8)	208 (40.9)	
None, animals must be free, n (%)	38 (7.7)	85 (16.7)	
Most important behavior performed by dairy cows:			<0.001[1]
Grooming, n (%)	106 (21.6)	70 (13.8)	
Maternal behavior, n (%)	263 (53.6)	340 (66.8)	
Reproduction, n (%)	62 (12.6)	33 (6.5)	
Game behavior, n (%)	60 (12.2)	66 (13.0)	
When milking, a dairy cow:			0.113[1]
Gets stressed, n (%)	275 (56.0)	254 (49.9)	
Is suffering, n (%)	42 (8.6)	60 (11.8)	
Is in pain, n (%)	88 (17.9)	88 (17.3)	
Has pleasure, n (%)	86 (17.5)	107 (21.0)	

[1] Chi-square test for independence

Table S6. Chilean university students´ perceptions toward animal welfare stratified by grade.

	**First** (n = 170)	**Second** (n = 209)	**Third** (n = 207)	**Fourth** (n = 174)	**Fifth** (n = 150)	**Sixth** (n = 90)	P-value
Importance of animal welfare in productive system, n (%)	165 (97.1)	203 (97.1)	204 (98.6)	173 (99.4)	141 (94.0)	88 (97.8)	0.063[1]
Quality of life in production of:							
Cow´s milk							
Good, n (%)	41 (24.1) ^{a}	38 (18.2) ^{a}	63 (30.4) ^{a}	51 (29.3) ^{a}	35 (23.3) ^{a}	27 (30.0) ^{a}	0.040[2]
Poor, n (%)	29 (17.1)	46 (22.0)	53 (25.6)	31 (17.8)	35 (23.3)	17 (18.9)	0.293[2]
Beef							
Good, n (%)	29 (17.1) ^{a}	43 (20.6) ^{a}	45 (21.7) ^{a,b}	34 (19.5) ^{a}	38 (25.3) ^{a,b}	34 (37.8) ^{b}	0.004[2]
Poor, n (%)	45 (26.5) ^{a}	43 (20.6) ^{a,b}	37 (17.9) ^{a,b}	42 (24.1) ^{a,b}	31 (20.7) ^{a,b}	8 (8.9) ^{b}	0.025[2]
Salmon							
Good, n (%)	3 (1.8)	8 (3.8)	12 (5.8)	5 (2.9)	5 (3.3)	4 (4.4)	0.444[1]
Poor, n (%)	48 (28.2) ^{a,b}	65 (31.1) ^{a}	47 (22.7) ^{a,b}	28 (16.1) ^{b}	38 (25.3) ^{a,b}	28 (31.1) ^{a,b}	0.013[2]
Pig							
Good, n (%)	54 (31.8) ^{a,c}	78 (37.3) ^{a}	48 (23.2) ^{c,b}	51 (29.3) ^{a}	32 (21.2) ^{a,b}	8 (8.9) ^{b}	<0.001[2]
Poor, n (%)	11 (6.5)	10 (4.8)	20 (9.7)	22 (12.6)	15 (10.0)	11 (12.2)	0.069[2]
Laying hens							
Good, n (%)	43 (25.3)	42 (20.1)	39 (18.8)	33 (19.0)	40 (26.7)	17 (18.9)	0.304[2]
Poor, n (%)	37 (21.8)	45 (21.5)	50 (24.2)	51 (29.3)	31 (20.7)	26 (28.9)	0.325[2]
Most important operations in animal production							0.082[1]
Food, n (%)	24 (14.1)	44 (21.1)	36 (17.4)	42 (24.1)	30 (20.0)	30 (33.3)	
Transportation, n (%)	7 (4.1)	7 (3.3)	5 (2.4)	5 (2.9)	2 (1.3)	2 (2.2)	
Slaughter, n (%)	11 (6.5)	12 (5.7)	14 (6.8)	13 (7.5)	11 (7.3)	8 (8.9)	
Rearing, n (%)	81 (47.6)	92 (44.0)	107 (51.7)	74 (42.5)	83 (55.3)	35 (38.9)	
Accommodation, n (%)	47 (27.6)	54 (25.8)	45 (21.7)	40 (23.0)	24 (16.0)	15 (16.7)	
Food products made while taking into account animal welfare compared to products that do not take this into account							0.159[2]
Better flavour, n (%)	91 (53.5)	112 (53.6)	121 (58.5)	94 (54.0)	83 (55.3)	42 (46.7)	
Same flavour, n (%)	28 (16.5)	38 (18.2)	35 (16.9)	25 (14.4)	35 (23.3)	25 (27.8)	
I don´t know, n (%)	51 (30.0)	59 (28.3)	51 (24.6)	55 (31.6)	32 (21.3)	23 (25.6)	
Food products made while taking into account animal welfare compared to products that do not take this into account							0.009[2]
Higher nutritional quality, n (%)	103 (60.6) ^{a}	111 (53.1) ^{a}	125 (60.4) ^{a}	115 (66.1) ^{a}	101 (67.3) ^{a}	46 (51.1) ^{a}	
Same nutritional quality, n (%)	29 (17.1) ^{a,b,c}	56 (26.8) ^{c}	35 (16.9) ^{a,b,c}	22 (12.6) ^{b}	20 (13.3) ^{a,b}	25 (27.8) ^{a,c}	
I don´t know, n (%)	38 (22.4) ^{a}	42 (20.1) ^{a}	47 (22.7) ^{a}	37 (21.3) ^{a}	29 (19.3) ^{a}	19 (21.1) ^{a}	

[1] Fisher´s exact test and posthoc test with Bonferroni corrections for multiple comparisons.
Different superscript letters indicate a significant difference.
[2] Chi-square test for independence and posthoc test with Bonferroni corrections for multiple comparisons.
Different superscript letters indicate a significant difference.

Table S7. Chilean university students´ perceptions toward dairy cows welfare stratified by grade.

	**First** (n = 170)	**Second** (n = 209)	**Third** (n = 207)	**Fourth** (n = 174)	**Fifth** (n = 150)	**Sixth** (n = 90)	P-value
Highest level of animal welfare for dairy cows in:							0.002[1]
In-door production, n (%)	3 (1.8) [a]	1 (0.5) [a]	7 (3.4) [a]	1 (0.6) [a]	4 (2.7) [a]	2 (2.2) [a]	
Grazing production, n (%)	78 (45.9) [a]	91 (43.5) [a]	91 (44.0) [a]	74 (42.5) [a]	62 (41.3) [a]	25 (27.8) [a]	
Mixture of in-door and grazing, n (%)	57 (33.5) [a]	93 (44.5) [a]	84 (40.6) [a]	84 (48.3) [a,b]	63 (42.0) [a]	57 (63.3) [b]	
None, animals must be free, n (%)	32 (18.8) [a]	24 (11.5) [a]	25 (12.1) [a]	15 (8.6) [a]	21 (14.0) [a]	6 (6.7) [a]	
Most important behavior performed by dairy cows:							0.004[1]
Grooming, n (%)	23 (13.5) [a]	33 (15.8) [a,b]	34 (16.4) [a,b]	31 (17.8) [a,b]	29 (19.3) [a,b]	26 (28.9) [b]	
Maternal behavior, n (%)	118 (69.4)[a]	141 (67.5) [a]	123 (59.4) [a,b]	96 (55.2) [a,b]	77 (51.3) [b]	48 (53.3) [a,b]	
Reproduction, n (%)	14 (8.2) [a]	17 (8.1) [a]	16 (7.7) [a]	19 (10.9) [a]	23 (15.3) [a]	6 (6.7) [a]	
Game behavior, n (%)	15 (8.8) [a]	18 (8.6) [a]	34 (16.4) [a]	28 (16.1) [a]	21 (14.0) [a]	10 (11.1) [a]	
When milking, a dairy cow:							<0.001[1]
Gets stressed, n (%)	81 (47.6) [a,b]	98 (46.9) [b]	119 (57.5) [a,b]	95 (54.6) [a,b]	94 (62.7) [a]	42 (46.7) [a,b]	
Is suffering, n (%)	26 (15.3) [a]	22 (10.5) [a,b]	19 (9.2) [a,b]	19 (10.9) [a,b]	14 (9.3) [a,b]	2 (2.2) [b]	
Is in pain, n (%)	26 (15.3) [a]	40 (19.1) [a]	40 (19.3) [a]	40 (23.0) [a]	16 (10.7) [a]	14(15.6) [a]	
Has pleasure, n (%)	37 (21.8) [a,b,c,d]	49 (23.4) [c,d]	29 (14.0) [b,d]	20 (11.5) [b]	26 (17.3) [b,d]	32 (35.6) [a,c]	

[1] Fisher´s exact test and posthoc test with Bonferroni corrections for multiple comparisons. Different superscript letters indicate a significant difference.

FULL SURVEY

SECTION I : SOCIODEMOGRAPHIC CHARACTERISTICS

1. Gender

() Female

() Male

2. Age

() 18-20

() 21-23

() 24-26

() More than 26

3. In which university do you study?

( ) Pontifical Catholic University of Chile

() University of Chile

4. Year of career

( ) First

() Second

( ) Third

( ) Room

() Fifth

() Sixth

5 . Study area

() Agricultural

() Art and architecture

() Exact and natural sciences

( ) Social Sciences

( ) Law

() Humanities

() Education

( ) Technology

( ) Health

() Administration and commerce

6. Commune of residence:

7 . It has some kind of relationship with the field

( ) No

() Family members living in the countryside

() Family member working in the agricultural sector

( ) Residential area

SECTION II: KNOWLEDGE

8 . Have you heard about animal welfare?

() Yes

( ) No

9 . Do you consider animal welfare an important issue in productive systems?

() Yes

() No

10 . What do you think is your level of knowledge about animal welfare?

() High knowledge

() Medium knowledge

() Low knowledge

() NO knowledge

( ) I'm not interested

1 1 . Please select from which source did you get your knowledge on animal welfare

( ) Educational institutions

() Social networks (facebook , twitter, instagram , etc.)

() Comments from friends and siblings

() Internet (easy to use search engines like google, wikipedia , etc.)

() Mass media (press, television, radio, cinema, etc.)

1 2 . What do you understand by animal welfare ?

() Adequate mental and physical state of an animal allowing the expression of their natural behaviors.

() Appropriate policies, legislation and standards developed and implemented in favor of animals.

() Avoid the use of animals to meet the needs and requirements of human beings.

1 3 . Have you heard about the five freedoms of animal welfare?

() Yes

() No

1 4 . Is there a current Law on Protection Animal in Chile?

( ) Yes

() No

( ) I do not know

1 5 . Do you think animals have emotions?

() Yes

( ) NO

( ) I do not know

1 6 . Do you think animals have different personality types?

() Yes

( ) NO

( ) I do not know

A then a series of questions about specific dealings will be made in milk production

1 7 . Does a dairy cow feel more comfortable walking on a smooth concrete floor or on land?

() Smooth cement

() Soil (in summer it is dry and in winter there is rain)

( ) I do not know

1 8 . Castration (extraction of the testicle) is a common management in productive systems. Is this practice done with the use of anesthetics (chemical that causes temporary loss of sensation of a part of the body)?

( ) True

( ) False

( ) I do not know

1 9 . Is it a common practice in dairy cows in the country separating the calf from the mother after birth?

( ) True

() False

( ) I do not know

20 . The dehorning is done in bovine dairies to calves to prevent the growth of a future horn. Is or is mainly done by:

( ) Esthetic

() Ease of handling

( ) I do not know

2 1 . Would you like an animal welfare course available for all faculties at your university ?

() Yes

( ) NO

SECTION III PERCEPTION

2 2 . In which of the following production systems do you believe that animals have a better quality of life?

() Cow's milk production system

() Beef production system

() Salmon production system

() Pig production system

() Laying hens production system

2 3 . In which of the following production systems do you believe that animals have a worse quality of life?

() Cow's milk production system

() Beef production system

() Salmon production system

() Pig production system

() Laying hens production system

2 4 . Under what conditions is the highest level of animal welfare offered for dairy cows?

() In-door production system (roofed housing)

() Grazing production system (trees and shrubs)

() A mixture of both

() None since they must be free

25 . Which of the following operations corresponds to the most important in an animal production system from your point of view?

() Food

() Transportation

() Slaughter

() Rearing (man power used for rearing animals from birth to weaning)

( ) Accommodation

26 . In your opinion, which is the most important behavior to be performed by dairy cows.

() Grooming (self-care)

() Maternal behavior (mother-child interaction)

() Sexual behavior (reproduction)

() Game behavior (entertainment)

27. From the physiological point of view, milking in a dairy cow corresponds to an event that causes:

() Stress

() Suffering

( ) Pain

() Pleasure

28. In your opinion, products made under animal welfare in terms of taste are:

() Food with better flavor

() Food with the same flavor as products that are not under Animal welfare

( ) I do not know

2 9 . In your opinion, products made under animal welfare in nutritional terms are:

() Foods with higher nutritional quality

() Foods of the same nutritional quality as foods without Animal welfare

( ) I do not know

Thank you for your participation!

https://es.surveymonkey.com/r/bienestaranimalycomunidaduniversitaria
